# A Comparative Analysis of Methods for Evaluation of ECG Signal Quality after Compression

**DOI:** 10.1155/2018/1868519

**Published:** 2018-07-18

**Authors:** Andrea Němcová, Radovan Smíšek, Lucie Maršánová, Lukáš Smital, Martin Vítek

**Affiliations:** ^1^Department of Biomedical Engineering, The Faculty of Electrical Engineering and Communication, Brno University of Technology, Technická 12, 616 00 Brno, Czech Republic; ^2^Institute of Scientific Instruments, The Czech Academy of Sciences, Královopolská 147, 612 64 Brno, Czech Republic

## Abstract

The assessment of ECG signal quality after compression is an essential part of the compression process. Compression facilitates the signal archiving, speeds up signal transmission, and reduces the energy consumption. Conversely, lossy compression distorts the signals. Therefore, it is necessary to express the compression performance through both compression efficiency and signal quality. This paper provides an overview of objective algorithms for the assessment of both ECG signal quality after compression and compression efficiency. In this area, there is a lack of standardization, and there is no extensive review as such. 40 methods were tested in terms of their suitability for quality assessment. For this purpose, the whole CSE database was used. The tested signals were compressed using an algorithm based on SPIHT with varying efficiency. As a reference, compressed signals were manually assessed by two experts and classified into three quality groups. Owing to the experts' classification, we determined corresponding ranges of selected quality evaluation methods' values. The suitability of the methods for quality assessment was evaluated based on five criteria. For the assessment of ECG signal quality after compression, we recommend using a combination of these methods: PSim SDNN, QS, SNR1, MSE, PRDN1, MAX, STDERR, and WEDD SWT.

## 1. Introduction

The evaluation of the quality of electrocardiogram (ECG) after compression is an essential part of compression in the broadest sense. Compression reduces the amount of data, which facilitates signal archiving, speeds up signal transmission (especially important in telemedicine), and reduces energy consumption. On the other hand, compression usually results in loss of signal quality. This arises in the case of lossy compression, which is the most commonly used technique because of its high efficiency. Indeed, while the quality of the signal after* lossless *compression is preserved, the efficiency is low. The aim of compression is to maximize the reduction of data amount while preserving the quality (diagnostic information). This naturally results in a compromise between efficiency and quality. It is thus desirable to express the compression performance through both efficiency and quality to avoid misunderstanding [1].

For the subsequent ECG analysis, the compressed signal should be of sufficient quality to ensure the avoidance of misdiagnosis. The quality can vary according to the aim of the analysis (e.g., specifying QRS complex morphology, tracking ST segment changes, and determining heart rate). For example, if we want to determine heart rate only, we can do this from a signal with lower quality and can therefore compress the signal much more than in case of, for example, tracking ST segment changes. Generally speaking, the quality of the signal after compression should be quantified to decide whether the signal is appropriate for further specific analysis or not [2]. For this purpose, it is advantageous to use automatized methods. These methods will facilitate the work of cardiologists and other medical staff since they will not have to deal with whether the signal is of sufficient quality or not [3]. This can save time [3], especially for the staff employed in telemedicine, where large amounts of data are transmitted and analysed. Quality indexes (the products of automatized algorithms) are also used for compression control [4]. If the signal is of low quality, it is compressed again with different settings. While existing literature presents various approaches (indexes and algorithms), there is no existing standardization or unification [3].

ECG compression is not yet commonly used in practice. This is because of the lack of reliable methods for an evaluation of signal quality after compression [5]. Indeed, the evaluation of the quality of ECG signal after compression is still an open and challenging problem [6]. One of the barriers here relates to the fact that various compression algorithms can cause various types of distortion (more or less important). This can be a major problem, especially in the case of evaluating algorithms without diagnostic information (e.g., Percentage Root mean square Difference (PRD)). Here, for one compression algorithm the achieved PRD can be, for example, 5 % and the important parts of the ECG signal are distorted (the compression algorithm for example distorts the ST segment). For another compression algorithm, the achieved PRD could be, for example, 10 % on the same ECG signal. But in this case mainly the noise is reduced and diagnostically important parts are not distorted. In other words, reduction of noise (as a secondary feature of good compression algorithms) causes the increase of PRD, but diagnosing will be simpler [7].

The main aims of this work are (1) to create a review of the methods for the evaluation of signal quality after compression and (2) to create recommendations regarding which quality metrics are the most suitable and what their threshold values must be to ensure that the signal is of sufficient quality. For the purpose of testing various quality measures, the signals from the second most cited standard database of ECG signals [8], the Common Standard in Electrocardiography (CSE) database [9], are used. These signals are compressed using an advanced and very popular wavelet-based Set Partitioning in Hierarchical Trees (SPIHT) algorithm [10, 11].

All the known (to the best of our knowledge) methods for the assessment of ECG signal quality after compression are described in Section 3 of this review along with their relative popularity (according to the number of citations). These methods were tested in terms of their suitability for the evaluation of the quality of ECG signal after compression. A description of the testing and its results including recommendations for the evaluation of ECG signal quality after compression are introduced in Sections 4 and 5, respectively. However, the efficiency of compression should be evaluated first, because this interacts with quality of compression. For this purpose, several metrics are used and are briefly described in Section 2.

## 2. Known Methods for Evaluation of Compression Efficiency

As mentioned above, compression results in a compromise between efficiency and quality. Hence, it is necessary to calculate both of them [12]. Three main algorithms are used for the evaluation of compression efficiency: compression ratio (CR); compression factor (CF) and average value length (avL). However, the existing literature is not consistent in the use of the terms CR and CF. In some sources, the authors use the term CR for (3) rather than CF ([5, 6, 13–21]), while in others ([22]), the CR is defined differently, that is, (2). Equations (1) and (3) below come from [23]. Using these equations, CR should be less than 1 and CF greater than 1 in order to reach compression. CF is the reciprocal value of CR.

(1) CR−=size  of  the  output  streamsize  of  the  input  stream

(2) CR2%=size  of  the  input  stream−size  of  the  output  streamsize  of  the  input  stream×100

(3) CF−=size  of  the  input  streamsize  of  the  output  stream

Equation (3) is also valid for another two similar methods, the sample reduction ratio (SRR) [6] and sample compression ratio (SCR) [24]. These methods differ from CF only in the fact that the numerator as well as denominator are expressed in samples (not bits). In [25], the bit compression ratio (BCR) is described, which is CF with both numerator and denominator which are expressed in bits (ideal case).

Data volume saving (DS) [26] is the measure which uses CF. This is shown in (4)

(4) $$\begin{array}{c} DS\left(\;\%\;\right)=100\times \left(\;1-\frac{1}{CF}\;\right) \end{array}$$

An alternative to CR and CF is avL [5] or average code length (ACL) [21]. These are two terms for one method shown in (5). It informs us about the number of bits that are used for coding one sample. From this the unit bits per sample (bps) follows.

(5) $$\begin{array}{c} avL\left(\;bps\;\right)=\frac{bit\;size\;of\;the\;output\;stream}{length\;of\;the\;signal\;in\;\;samples} \end{array}$$

CF can be calculated from avL as a ratio between the original resolution of the signal (in bps) and the avL [27].

Another method similar to avL is called compressed data rate (CDR) [6, 13, 20]. This method can be calculated using different variables (as shown in (6) and (7)) and informs us how many bits are needed for coding one second of signal. Resolution in (7) expresses number of bits per sample of the original signal and CF is calculated in bits. The unit has the same abbreviation as avL (bps) but means bits per second (therefore it is not abbreviated here). Bit rate [5, 21] is very similar to CDR, but it does not work with sampling rate, as is shown in (8).

(6) CDR  bits  per  second=sampling  rate×bit  size  of  output  streamduration  of  the  signal

(7) CDR  bits  per  second=sampling  rate×resolutionCF

(8) bit  rate  bits  per  second=bit  size  of  output  streamduration  of  the  signal

The variables “size of the input stream” and “size of the output stream” are usually not clear, while in an ideal case, it is the bit size (this means number of bits before/after signal compression). In other instances, it can be the length of the signals (number of samples) or the size of the output file (depends on file format, e.g., *∗*.txt, *∗*.mat, *∗*.zip, and its compression algorithm if it exists). Therefore, we use avL in this work, because it has clear units. The avL method is, moreover, simply comparable with the original bit resolution of the signal (usually 8-16 bps [6, 28–30] depending on the recording device).

## 3. Known Methods for Evaluation of the ECG Signal Quality after Compression

For the evaluation of ECG signal quality after compression and reconstruction, various methods are used. These methods can be divided into two main groups: subjective methods and objective methods. Subjective methods are based on the assessment of ECG signal quality by cardiologists or other experts and are described in Section 3.1, while objective methods are further divided into the following: methods without diagnostic information, methods with diagnostic information based on wavelet transform (WT), and methods with diagnostic information based on delineation. These three groups of methods are defined in Sections 3.2.1, 3.2.2, and 3.2.3, respectively. While the latter two groups of methods noted above both provide diagnostic information, their principles are completely different and are therefore described separately. There also exists at least one method for multilead ECG signal quality evaluation, which is briefly described in Section 3.2.4. In Section 3.2.5 single-lead ECG quality assessment methods are briefly mentioned. A search involving all these methods was conducted through Scopus and they were subsequently sorted according to their popularity. In Section 3.2.6, the ten most commonly used methods are listed. In this review, we have unified the style of presenting the equations for the individual methods to minimise the reader's work.

### 3.1. Subjective Methods

The subjective methods for ECG quality evaluation are medically accepted [31], unlike the majority of the objective methods. However, a subjective evaluation of ECG signal after compression requires the input of experts or specialist cardiologists. Thus, the main disadvantage of these methods is that they require the time of such individuals and the attendant financial costs. Moreover, they can only be performed offline. There also exist the problems of intraobserver (one cardiologist can evaluate the signal differently at different times) and interobserver (two cardiologists can diagnose the same signal differently) variability in diagnosis [32]. The factors that can influence this include knowledge level, work experience, practices (procedures) of the particular clinic, motivation, mental fatigue, and psychological state.

The direct method involves the evaluation of the quality of the reconstructed signal visually [3, 33] by a cardiologist or a holter technician. This method can be used as a reference for objective methods. Several sources ([5, 13]) are stricter and insist that the quality should always be verified by a cardiologist. Indeed, these authors [13] suggest that even if the signal is appropriate for further analysis in terms of objective methods, it should be evaluated subjectively by a physician.

The second subjective method is based on the difference in diagnosis from the original and compressed ECG [32, 34]. However, the gold standard of the methods for the evaluation of the ECG signal quality after compression is the Mean Opinion Score (MOS) test [2, 3, 28, 31, 35, 36]. Its output is MOS_error_, which informs us directly about the diagnostic distortion (in %). The MOS test consists of blind and semiblind tests. In [35] the MOS test was completed by three cardiologists, while in [30] three cardiologists and three researchers participated. In the blind test, they evaluated the general quality score (from 1, very bad, to 5, excellent) and the interpretation of P wave, QRS complex, T wave, ST segment, and abnormal beats (scales 1-8). In the semiblind test, they assessed the similarity between the original and reconstructed signals (from 1, completely different, to 5, identical) and binary evaluated whether they were diagnosed differently without access to the details of the original signal (for more detailed information on this see [35]). In [37], the authors defined four signal quality groups according to MOS_error_: very good (0-15 %), good (15-35 %), not good (35-50 %), and bad (> 50 %).

### 3.2. Objective Methods

Objective methods are based on mathematical equations and have no need for expert human interaction (except for the developing phase of some algorithms, where the cardiologist can, e.g., set the weighting matrix or select appropriate features). These methods are automatized and save time and the costs of the cardiologists. By using these methods, we also avoid the intraobserver and interobserver variability. While the majority of these methods can work online or with some buffer, they should be carefully selected based on their performance, if they are to be used instead of subjective methods. Indeed, as explained below, not all the methods are suitable for ECG quality assessment. Generally speaking, they are based on various principles and can be calculated for the whole signal or in a time window. Some of the methods also include diagnostic information, while these methods are more complex and nontrivial. Some also require a delineation algorithm to assess the diagnostic quality. However, no perfect, universal delineation algorithm currently exists, which can lead to inaccurate values of diagnostic distortion. The objective method is regarded as “good”, provided it corresponds with the subjective evaluation of the cardiologist [28]. In this chapter, more than 40 objective methods are described.

#### 3.2.1. Without Diagnostic Information

Subjective methods without diagnostic information are easy to compute and are therefore popular (they are cited 619 times according to Scopus). Many authors use these methods, meaning new results can be compared with theirs. However, they do not have such high informative value as methods* with *diagnostic information. In fact, their values can be more or less dependent on the voltage of the signal. To avoid this dependence on voltage, normalization is carried out [2]. The baseline fluctuations influence the output value of these methods [35], and, ideally, they should be removed [36]. Meanwhile, some methods are dependent on the noise level [13]. While the equations are identical for all the various parts of the ECG signal, not all of them have equal importance from a diagnostic point of view [35]. For example, according to [32], higher distortion of the QRS complex can be less important than lower distortion of the baseline, where the problem with P wave detection can arise. The correlation between the objective methods without diagnostic information and diagnostic distortion is quite weak [28], which is the main disadvantage of these methods.

Error signal *e*(*n*) [1, 16] is probably the simplest way to compare the original and reconstructed signal (numerically or visually).

(9) $$\begin{array}{c} e\left(\;n\;\right)=x\left(\;n\;\right)-\tilde{x}\left(\;n\;\right) \end{array}$$

where *x*(*n*) is the original signal, $\tilde{x}(n)$ is the reconstructed signal, and *n* is the index of each sample of the signal of length *N*.

In [38] a similar measure is published. It is called Local Absolute Error (LAE) and applies absolute value on (9).

Mean Square Error (MSE) [2, 6, 13, 28] is computed according to

(10) $$\begin{array}{c} MSE\left(\;{mV}^{2}\;\right)=\frac{1}{N}\cdot \overset{N}{\underset{n=1}{\sum }}{\left[\;x\left(\;n\;\right)-\tilde{x}\left(\;n\;\right)\;\right]}^{2} \end{array}$$

and its normalized version MSE (NMSE) [13, 28] according to

(11) $$\begin{array}{c} NMSE\left(\;-\;\right)=\frac{\sum_{n=1}^{N}{\left[x\left(n\right)-\tilde{x}\left(n\right)\right]}^{2}}{\sum_{n=1}^{N}{\left[x\left(n\right)\right]}^{2}} \end{array}$$

Root Mean Square Error abbreviated as RMS in [3, 28, 35, 39] or abbreviated as RMSE in [2, 13, 38, 40] is mathematically described by (12). In [20, 41] the equation of RMS differs in terms of subtracting 1 in denominator (13).

(12) RMS1mV=∑n=1Nxn−x~n2N

(13) RMS2mV=∑n=1Nxn−x~n2N−1

The advantage of this method is preserving of original unit (millivolts) [40]. Using RMS as a quality measure for control of the compression is supposedly more effective than using PRD [40].

Normalized version of RMS (NRMSE) [2, 13, 28] is shown in (14). NRMSE is almost identical with following PRD except for the multiplication of 100 % (in case of PRD).

(14) $$\begin{array}{c} NRMSE\left(\;-\;\right)=\sqrt{\frac{\sum_{n=1}^{N}{\left[x\left(n\right)-\tilde{x}\left(n\right)\right]}^{2}}{\sum_{n=1}^{N}{\left[x\left(n\right)\right]}^{2}}} \end{array}$$

The Percentage Root mean square Difference (PRD) [3, 5, 10, 16–21, 26, 28, 33, 35, 39, 41–43] or Percentile Root Mean Square Difference (PRMSD) [38] takes into account mean of the signal (DC component) and the offset (constant value which is added to signal for storing purposes; e.g., 1,024 for MIT-BIH Arrhythmia Database [13, 44, 45]. Both methods have the same equation:

(15) $$\begin{array}{c} PRD\left(\;\%\;\right)=\sqrt{\frac{\sum_{n=1}^{N}{\left[x\left(n\right)-\tilde{x}\left(n\right)\right]}^{2}}{\sum_{n=1}^{N}{\left[x\left(n\right)\right]}^{2}}}\cdot 100 \end{array}$$

It is evident that PRD and NRMSE differ only in terms of multiplying the former by 100, which means the units of PRD are a percentage. For further analysis, the NRMSE is redundant. If the signal has a DC component and/or the nonzero offset and the PRD is calculated, its value will be artificially lower [36]. PRD will be also lower in the case of high standard deviation of the signal [2]. In [40] it is shown that the PRD does not correspond to the error signal. Therefore, the normalized version is used.

PRD has three normalized versions (PRDN) [2, 5, 13, 15, 18–20, 24, 28, 35, 36, 41–43, 46, 47] that should be used if the signal has a nonzero DC component and/or an added offset (see (16), (17), and (18)).

(16) PRDN1%=∑n=1Nxn−x~n2∑n=1Nxn−x−2·100

(17) PRDN2%=∑n=1Nxn−x~n2∑n=1Nxn−x−−P2·100

(18) PRDN3%=∑n=1Nxn−x~n2∑n=1Nxn−P2·100

where $\overset{-}{x}$ is the mean of the original signal (the DC component) and *P* is the offset. If these components are subtracted from the signal correctly, the results of PRD and PRDN are the same. It is very important to distinguish between PRD and PRDN. Many authors do not define the type of PRD that they use and/or do not mention whether the DC component and offset were removed. Therefore, it is not possible to compare the performance of such compression algorithms properly [43]. PRDN has higher value than PRD if the signals contain DC component and/or an offset [28]. After removing the offset, the DC component is still nonzero [1]. Therefore, the PRDN1 measure is the correct one [1], because it eliminates both in one step. Noise and DC component have no diagnostic meaning [6].

According to [13, 35, 42], signals of “very good” and “good” quality have PRDN1 and PRDN2 less than 9 % for specific compression algorithms. The specific threshold value of PRDN depends on the principle of compression.

PRD or PRDN can be also calculated for each subband of signal after wavelet transform [3]. According to [12], another variant of PRD (here marked as PRDT) can be calculated using (19). The difference is that PRDT uses coefficients of the wavelet transform instead of the samples of the original signal.

(19) $$\begin{array}{c} PRDT\left(\;-\;\right)=\sqrt{1-\frac{\sum_{q\in Q}^{\;}{\left|c_{q}\right|}^{2}}{\sum_{q}^{\;}{\left|c_{q}\right|}^{2}}} \end{array}$$

where* q* are the indexes of all wavelet coefficients, *Q* is the set of indexes of the most significant coefficients left after compression, and *c* are the transform coefficients.

The Moving Average PRD (MAPRD) [12] was created as a local measure. MAPRD calculates the amount of distortion in a sliding window. Equation (20) is for one window of length *w*.

(20) $$\begin{array}{c} MAPRD\left(\;-\;\right)=\sqrt{1-\frac{\sum_{n=1}^{w}{\left[\tilde{x}\left(n\right)\right]}^{2}}{\sum_{n=1}^{w}{\left[x\left(n\right)\right]}^{2}}} \end{array}$$

Signal to Noise Ratio (SNR) [2, 3, 19, 20, 28, 35, 41] corresponds to (21). Noise is here understood as a difference between original and reconstructed signal (error in (9)).

(21) $$\begin{array}{c} SNR1\left(\;db\;\right)=10\cdot \log_{10}\left(\;\frac{\sum_{n=1}^{N}{\left[x\left(n\right)-\overset{-}{x}\right]}^{2}}{\sum_{n=1}^{N}{\left[x\left(n\right)-\tilde{x}\left(n\right)\right]}^{2}}\;\right) \end{array}$$

SNR can be also computed with use of PRD.

(22) SNR2db=−20·log10⁡0.01·PRD=40−20·log10⁡PRD

SNR is more accurate than PRD and PRDN measures when compared with MOS [28].

Peak Signal to Noise Ratio (PSNR) [18] is shown in

(23) PSNRdb=20·log10⁡max⁡xn1/N·∑n=1Nxn−x~n2

Maximum Amplitude Error (abbreviated as MAX [3, 35, 40] or MAE [38]), Peak Error (PE) [1–3, 13, 28, 35], Maximum Absolute Error [13], or Maximum Error (MaxErr) [15] is one measure, which informs us about local distortion of the signal and is usually calculated separately for each cycle [28] using

(24) $$\begin{array}{c} \text{MAX}\left(\;mV\;\right)=\underset{n}{\max}\left\{\;\left|\;x\left(\;n\;\right)-\tilde{x}\left(\;n\;\right)\;\right|\;\right\} \end{array}$$

It is possible to calculate MAX for the whole signal as a mean value of MAX in each cycle [28]. MAX can be also modified by weighting the error signal [28]. Every sample of the error signal is weighted by the absolute value or the energy value of the original sample.

Normalized Maximum Amplitude Error (NMAE) [43], in some sources ([2, 13, 28]) called NMAX, is shown in (25). It informs us about maximal distortion in the signal (maximally distorted sample). In some studies, (e.g., [38]), the equation does not include 100, and its units are not a percentage. Normalization lies in dividing the numerator by the difference between the maximum of *x*(*n*) and the minimum of *x*(*n*).

(25) $$\begin{array}{c} NMAX\left(\;\%\;\right)=100\cdot \frac{\max_{n}\left\{\left|x\left(n\right)-\tilde{x}\left(n\right)\right|\right\}}{\max_{n}\left\{x\left(n\right)\right\}-\min_{n}\left\{x\left(n\right)\right\}} \end{array}$$

Peak Amplitude Related Error (PARE) [38] is a normalized method and its product is the error signal (not only one number) as can be seen in

(26) $$\begin{array}{c} PARE\left(\;-\;\right)=\frac{x\left(n\right)-\tilde{x}\left(n\right)}{\max_{n}\left\{x\left(n\right)\right\}} \end{array}$$

Standard Error (S.E.) [13], STDERR [2, 28], or Standard deviation of Errors (StdErr) [15] is one method defined by two different equations. According to [2], the equation of StdErr is identical as (13) of RMS2. The authors in [13, 28] express the STDERR by

(27) $$\begin{array}{c} STDERR\left(\;mV\;\right)=\sqrt{\frac{1}{N-1}\cdot \overset{N}{\underset{n=1}{\sum }}{\left[e\left(n\right)-\overset{-}{e}\right]}^{2}} \end{array}$$

where* e* is the difference between the original and the reconstructed signal and $\overset{-}{e}$ is the mean value of* e*.

Cross Correlation (CC) [28, 47] or Normalized Cross Correlation (NCC) [2, 13] is defined according to

(28) CC1−=1/N·∑n=1Nxn−x−·∑n=1Nx~n−x~−1/N·∑n=1Nxn−x−2·1/N·∑n=1Nx~n−x~−2

where $\overset{-}{\tilde{x}}$ is the mean value of the reconstructed signal. To set the record straight, (28) of CC1 is incorrect. The right form is (29) [3, 14, 43, 47].

(29) $$\begin{array}{c} CC2\left(\;-\;\right)=\frac{\sum_{n=1}^{N}\left[x\left(n\right)-\overset{-}{x}\right]\cdot \left[\tilde{x}\left(n\right)-\overset{-}{\tilde{x}}\right]}{\sqrt{\sum_{n=1}^{N}{\left[x\left(n\right)-\overset{-}{x}\right]}^{2}}\cdot \sqrt{\sum_{n=1}^{N}{\left[\tilde{x}\left(n\right)-\overset{-}{\tilde{x}}\right]}^{2}}} \end{array}$$

Percentage area difference (PAD) [2, 24, 43] is shown in

(30) PAD%=∫titfxtdt−∫titfx~tdttf−ti·maxn⁡xn⁡−minn⁡xn·100

where *t*_*i*_ and *t*_*f*_ are the times of the beginning and the end of the segment of interest.

Quality coefficient (*κ*) [48] is introduced in

(31) $$\begin{array}{c} \kappa \left(\;-\;\right)=\frac{1}{N}\cdot \overset{N}{\underset{n=1}{\sum }}{\left[\;\frac{x\left(n\right)-\tilde{x}\left(n\right)}{\left(1/N\right)\cdot \sum_{n=1}^{N}\left|x\left(n\right)\right|}\;\right]}^{2} \end{array}$$

The same source [48] introduces another similar measure: method of averaged interval. The quality coefficient *κ* is computed for the intervals of required length (the authors use the length of 25 samples); the result is their average.

The method angle between two vectors (*α*) [48], shown in (32), is based on the fact that the dot product of orthogonal signals is zero.

(32) $$\begin{array}{c} \cos\alpha =\frac{x\cdot \tilde{x}}{\left\|x\right\|\cdot \left\|\tilde{x}\right\|}=\frac{\sum_{n=1}^{N}\left|x\left(n\right)\cdot \tilde{x}\left(n\right)\right|}{\sum_{n=1}^{N}\left|x\left(n\right)\right|\cdot \sum_{n=1}^{N}\left|\tilde{x}\left(n\right)\right|} \end{array}$$

Quality score (QS) [5, 19, 20, 41] is a combination of two methods: CF as an efficiency measure and PRD as a measure of quality (see (33)).

(33) $$\begin{array}{c} QS\left(\;-\;\right)=\frac{CF}{PRD} \end{array}$$

QS is suitable for comparison of signals with various CF and PRD. The greater the QS is, the better the compression is.

#### 3.2.2. With Diagnostic Information Based on WT

These methods reflect the diagnostic information contained in the ECG signal. They inform about the distortion of, e.g., P wave, QRS complex, or T wave.

Percentage Error (PE) in [3] can be calculated according to (34) from the wavelet coefficients *c* and $\tilde{c}$ of the original and reconstructed signal, respectively. Index *i* is the *i*-th subband of WT.

(34) $$\begin{array}{c} PE\left(\;\%\;\right)=\frac{\sum \left|c_{i}-\tilde{c_{i}}\right|}{\sum \left|c_{i}\right|}\cdot 100 \end{array}$$

Wavelet-based Weighted PRD (WWPRD) [3, 28] is a method based on wavelet transform and weighting. The signal is decomposed into subbands using the wavelet transform (9-7 biorthogonal wavelet). The number of levels of WT is based on sampling frequency (for details see [3]). Then, the PRD is calculated for each subband similarly to (15); the only difference is in using wavelet subbands instead of the original signal. The use of nonnormalized PRD is relevant, because the means of the original and the reconstructed signal were subtracted before. There exist two types of weights: (1) heuristically set (WWPRD_h_), and (2) calculated as a Wavelet Subband Normalized Area (WWPRD WSNA). The second type of weights, WSNA, takes into consideration the amplitudes and shapes of the signal components. They are calculated as a sum of the wavelet coefficients in the respective subband divided by the sum of all wavelet coefficients (in all subbands). Here, we will consider only WWPRD WSNA (shortly only WWPRD), because the weights can be precisely calculated. The WWPRD value is calculated as a sum of the weighted PRDs calculated in individual subbands. According to [3], this method outperforms PRD, PRDN1, SNR, PE, CC, and RMS in terms of accuracy/uncertainty (in comparison with MOS). However, the tables and graphs [3] show that the CC has even higher accuracy than WWPRD, according to the provided statistical analyses. Indeed, WWPRD can be affected by baseline wandering [39]; therefore it should be eliminated. Based on cardiologists' verification of compressed signals, the authors of [39] recommend compressing ECG signals with a WWPRD under 10 %.

Wavelet-Energy based Weighted PRD (WEWPRD) [4] and Wavelet-Energy based Diagnostic Distortion (WEDD) [28, 30, 46] are two names for one method based on WT and weighting (from now abbreviated as WEDD). The signal is decomposed into subbands using the WT. Based on the knowledge of the energy contribution of each frequency subband, the weight for each subband is calculated. Next, the PRD is calculated for each subband similarly to (15); the only difference is in using wavelet subbands instead of the original signal. The WEDD for each subband is then calculated as a product of its PRD and weight. The final WEDD for one ECG signal is then obtained as a sum of the WEDDs of all subbands. WEDD was used for control of the SPIHT compression algorithm [4]. It optimizes the rate-distortion performance better than PRDN and WWPRD [46]. WEDD is also robust to the presence of noise in the signals, while it is sensitive to any distortion of P waves, T waves, and QRS complex [28, 46]. Overall, The WEDD algorithm outperforms PRD and WWPRD [46].

Based on an adjusted MOS method, five quality groups of the signal were determined [3]: excellent; very good; good; not bad; and bad. Based on the values of WWPRD [3], PRD [3], and WEDD [28] it can be predicted in which group the ECG signal belongs. The highest mean correct prediction value (95 %) and the lowest normalized prediction error (0.6876 %) have the WEDD measure [28]. In other words, by using the WEDD measure, the signal can be classified into one of the five quality groups with the lowest error among all available methods.

Multiscale Entropy-based Weighted PRD (MSEWPRD) [31] is the newest alternative to WWPRD and WEDD. It is also based on decomposition of the signal using the WT and weighting. The procedure of decomposition and PRD calculation is identical to that of both the previous methods. The innovation here lies in the different calculation of weights, which is based on multiscale entropy calculated in each subband. There exist three methods for weight estimation: WSNA; Relative Wavelet Subband Energy estimation (RWSE); and Relative Mean Wavelet Subband Energy estimation (RMWSE). RWSE enhances lower subbands (higher energy) and while RMWSE also enhances lower subbands, it also suppresses higher subbands. Meanwhile, MSEWPRD using RMWSE results in the highest correlation with the subjective measure MOS among the methods of PRD, WWPRD, WEDD, MSEWPRD WSNA, MSEWPRD RWSE, and MSEWPRD RMWSE [31]. Therefore, MSEWPRD is appropriate for quality evaluation of noisy ECG signals.

#### 3.2.3. With Diagnostic Information Based on Delineation

The methods with diagnostic information based on delineation have the most predictive value. However, their disadvantage is the computational complexity and, for some, the presence of a cardiologist while developing and setting the algorithm (e.g., for feature selection or weights setting). The accuracy of these methods depends on the accuracy of the delineation algorithms. It is thus necessary to use accurate and robust delineation algorithms.

Weighted PRD (WPRD) [2, 14] is an improved version of PRD and includes diagnostic information. As shown in (35), WPRD is the sum of separately calculated distortions of P wave, Q wave, QRS complex, and ST segment. Furthermore, each distortion is weighted in terms of importance of the wave or complex. The weights should be determined by a cardiologist. The accuracy of the WPRD depends on the quality of delineation [28].

(35) $$\begin{array}{c} WPRD=\sqrt{\frac{\sum_{k=1}^{K}\omega_{k}\cdot \gamma_{k}}{\sigma }} \end{array}$$

where *ω*_*k*_ are the weights, *γ*_*k*_ is the RMSE of current wave/complex/segment, and *σ* is the power of the original signal ($\sqrt{\gamma /\sigma }$ is called PRD in [14]).

The Clinical Distortion Index (CDI) [3, 49] is based on features extraction and the comparison between the original and the reconstructed signal (see (36)). For the purpose of CDI calculation, 12 features were used among durations, amplitudes, and morphology. The features were weighted according to their clinical importance (see (37)).

(36) dkm=pkorigm−pkrecmVrefm

(37) CDIi−=diTEditrE

where* k* is the index of the heartbeat,* m* is the feature index (1 ≤ *m* ≤ *M*, where* M* is the number of clinical features),* V*^*ref*^ is the reference value for each feature, and *p*_*k*_^*orig*^(*m*) and *p*_*k*_^*rec*^(*m*) are the specific clinical features in the particular beat of original and reconstructed signal, respectively. The values of* V*^*ref*^ are determined by cardiologists and are stated in [49]. Meanwhile,* d* is the features vector,* E* is a diagonal weighting matrix (in [3, 49] the identity matrix is used), and tr (trace) is the sum of the elements on the main diagonal of the matrix.

For the WDD estimation [28, 30, 35, 36, 43, 50, 51], it is first necessary to delineate both original and reconstructed signal. Using the delineated points, 18 features among locations, durations, amplitudes, and the shapes of waves and complexes of the ECG signal are extracted and WDD is calculated according to

(38) $$\begin{array}{c} WDD\left(\;\beta ,\hat{\beta }\;\right)=\Delta \beta^{T}\cdot \frac{\Lambda }{tr\left[\Lambda \right]}\cdot \Delta \beta \cdot 100 \end{array}$$

where *β* is the diagnostic features of the original signal, $\hat{\beta }$ is the diagnostic features of the reconstructed signal,* Δβ* is the normalized difference vector, and* Λ* is a diagonal matrix of weights. The equation exists for a calculation of differences (distances) of durations and amplitudes [35]. Calculation of the shape features differences is based on a penalty matrix that is constructed with the use of a database of possible shapes created by a cardiologist. This method is the most complex one from all mentioned in this paper and more detailed information can be found in [35, 50]. The WDD correlates well with visual inspection [28] and also with MOS, more so, in fact, than PRD [35, 50]. On the other hand, the weights were set by noncardiologist for the purpose of study [35]. As is written in [35], the weights should reflect the clinical importance of used features in real world. Therefore, we suppose that, to reach the highest objectivity of the method, it requires the cooperation of cardiologist (to set the weights, which are clinically relevant) and is therefore quite expensive and time consuming (at least at the beginning).

Average absolute error (AAE) [47] is a method based on extraction of ten features among amplitudes, durations, and slope. Initially, the features are extracted in both original and reconstructed signal before the error for each feature within each cycle* k* is calculated:

(39) $$\begin{array}{c} \lambda_{k}\left(\;\%\;\right)=\left|\;\frac{p_{k}^{orig}-p_{k}^{rec}}{p_{k}^{orig}}\;\right|\cdot 100 \end{array}$$

At the end of the process, the errors of all features are averaged out for the whole signal. AAE was used for control of ECG signal compression based on discrete sinc interpolation [47].

The method based on heartbeats classification using multilayer perceptron neural networks (NN) [5] also belongs in this group of methods. Here, it is first necessary to segment the ECG signal to individual heartbeats using the R-wave detection algorithm. The heartbeats are further classified into eight groups (the eight most common types of heartbeats). NN is then trained on the original signal and tested on the reconstructed signal.

Another method for quality assessment of ECG signal after compression based on the sensitivity (SE) and specificity (SP) of QRS detection [26, 52] is introduced by (40) and (41). The authors use the equation for positive predictivity (+P) for calculation of specificity. The QRS complexes were detected in the reconstructed signal and their positions were compared with annotations. A tolerance of 88 ms on both sides was considered [52]. In [25] the authors use SE and +P with correct equations.

(40) SE%=TPTP+FN·100

(41) +P%=TPTP+FP·100

where* TP* (true positives) are correctly detected QRS complexes,* FN* (false negatives) are QRS complexes that were not detected, and* FP* (false positives) are QRS complexes that are incorrectly detected (according to annotations).

The percentage similarity (PSim) [26, 52] is a measure based on features derived from detected QRS complexes. Initially, the QRS complexes are detected, then the features (*p*) are calculated, mean normal-to-normal (NN), standard deviation of NN (SDNN), low-frequency/high-frequency (LF/HF) ratio using Lomb periodogram to compute the power spectral density for low frequencies (0.04-0.15Hz), and high frequencies (0.15-0.4Hz), and high-frequency (HF) power. A comparison of the features extracted from the original signal with features derived from reconstructed signal is performed according to

(42) $$\begin{array}{c} PSim\left(\;\%\;\right)=100-\left(\;\frac{\left|p^{orig}-p^{rec}\right|}{p^{orig}}\cdot 100\;\right) \end{array}$$

Similarity [26] is a method that also uses the detection of QRS complexes. The complexes are detected in the reconstructed signal and then compared with annotations of QRS complexes (e.g., from the standard databases) according to (43). From the corresponding article, it is not clear whether the authors consider only positions or both positions and values (amplitudes).

(43) Similarity%=100·annotatedValue−lossyValueannotatedValue

Heart rate trace (HRT) [26] is a calculation of heart rate in beats per minute (bpm) according to

(44) $$\begin{array}{c} HRT\left(\;bpm\;\right)=60\cdot \frac{fs}{beatIntervals} \end{array}$$

where* fs* is the sampling frequency of the signal and* beatIntervals* is the length of the RR interval in samples.

Detection of five ECG significant points is a basis of the method published in [7]. Here, the original signal and then the reconstructed signal are delineated. Then, the positions of significant points in both the original and the reconstructed signal are compared with annotated positions (considering tolerance). The method was tested on signals from the CSE database compressed with the SPIHT algorithm and the authors stated that the minimum acceptable avL was 0.8 bps, with PRDN at around 5 %.

Dynamic time warping (DTW) [53, 54] is a method that allows the aligning (warping) of two signals to reach the same length. If DTW is applied on both the original and the reconstructed signal, the fiducial points of the original signal should match the fiducial points in the reconstructed signal (considering tolerance). If they do, the reconstructed signal is of high quality and the diagnostic information is preserved. However, if the fiducial points do not match, the signal is distorted. In order to find the fiducial points, delineation algorithms are used. According to [53], this method provides similar information to that of a cardiologist. In fact, it states how the positions of the fiducial points differ, on average, in the original and reconstructed signals and what their standard deviation is. However, the method has not been described in detail.

Partial PRD [53, 54] is a method calculating PRD separately in diagnostically important segments of the ECG signal (PQRST complex, from the P onset to the T offset) and diagnostically unimportant segments between PQRST complexes (from the T offset to the P onset). The distortion in PQRST segments should be as low as possible, while the distortion of interbeat segments can be higher. The authors of this method used annotations of P onset and T offset, while in terms of testing the method, they used signals from a fully annotated QT Database.

#### 3.2.4. Methods Developed for Multilead ECG

The objective methods described above were primarily designed for single-lead ECG. For multilead ECG (MECG), indexes such as PRD, MSE, RMSE, WEDD, or WDD can be applied separately for each lead [2, 30]. To express the distortion of MECG with one single figure, the average value of the following measures along all leads can be calculated: multichannel PRD (MPRD); multichannel MSE (MMSE); multichannel RMSE (MRMSE); and multichannel WEDD (MWEDD) [2]. There also exist methods that were developed specifically for MECG.

MSD diagnostic measure is based on multivariate sample entropy (MSampEn), which is an alternative to single-lead sample entropy [2]:

(45) $$\begin{array}{c} MSD=e_{o}-e_{r} \end{array}$$

where* e*_*o*_ and* e*_*r*_ are the MSampEn values for original and reconstructed signal, respectively. The calculation of MSampEn is not trivial and it is explained in detail in [55].

#### 3.2.5. Single-Lead ECG Quality Assessment Methods

To make the picture complete, there also exist methods, which can assess the quality (clinical acceptability) of single ECG signal; it means without any reference (such as original signal in case of compression). The review of these methods is in [56, 57] which is an example of one of the latest methods. The signal is very often corrupted with some noise and artefacts. This fact can, e.g., make the diagnosing more difficult or even inaccurate and decrease the accuracy of detectors and delineation algorithms. It is good to know the quality of the signal (it is most often categorized into two groups, acceptable and unacceptable [56]). These methods are not directly connected with compression, but they can be utilized in this area. If the signal or its part is unacceptable, it is discarded and the compression and transmission from wearable sensors are not provided. Another possibility is to set compression algorithm adaptively based on the knowledge about its quality [56].

#### 3.2.6. Popularity of the Methods

The popularity of the methods for evaluation of ECG signal quality after compression was ascertained using Scopus. A search on articles that used specific methods was initiated using keywords and Boolean operators. In all cases, the keywords “ECG” and “compression” were used with the Boolean operator AND. Simultaneously, the whole name or abbreviation(s) of the quality evaluation method were used with the Boolean operator OR. One example of our use of keywords and Boolean operators is* TITLE-ABS-KEY (“ecg” AND “compression”) AND ALL (“weighted diagnostic distortion” OR “wdd”)*. The results of the search were manually corrected since some of the articles were deemed irrelevant. The ten most commonly used methods found are shown in Table 1.

From Table 1 it is clear that PRD and its variants are the most popular methods, while WDD, a complex method based on delineation, was second. Meanwhile, one of the methods based on wavelet transform (WEDD) is the eighth most cited, while around half of the investigated methods were mentioned only once or twice.

## 4. Materials and Methods

The methods described above were tested in terms of their suitability for the assessment of ECG quality after compression. We used a total of 1,875 (125 records, 15 leads) ECG signals from the CSE database, which is briefly described in Section 4.1. For the compression of ECG signals, we used an algorithm based on WT and SPIHT, and Section 4.2 deals with this. Compressed signals were also evaluated subjectively by two experts, who classified the signals into three quality groups. This is addressed in Section 4.3. Finally here, Section 4.4 describes the selection of the methods that were tested.

### 4.1. CSE Database

The CSE database [9] is the second most cited standard database [8]. Dataset 3 from the CSE database is used in this work for testing purposes. The dataset includes 125 original 15-lead ECG signals (12 standard leads and three Frank leads) [9]. These signals are ten seconds in length and were sampled at 500 Hz, while their resolution is 16 bps and the quantization level <= 5 *μ*V. The Frank leads of signals nos. 60, 68, 76, 84, 92, 100, 108, and 124 were excluded from further analysis because they are not correctly sensed [9]. One study [8] extended the annotations of the CSE database and thus extended the information on signals in terms of compression ability and the quality of the reconstructed signals. More information about the CSE database can be found in [8, 9]. The CSE database has no offset (like, for example, the MIT-BIH Arrhythmia Database has for storing purposes [44, 45]).

### 4.2. Compression Method Based on WT and SPIHT

All the signals from the CSE database are compressed using the algorithm based on WT (dyadic discrete time WT followed by dyadic decimation) and SPIHT. SPIHT is the progressive iterative compression algorithm. Its output is a bit flow, which can be stopped anytime. The algorithm can be controlled by the user and when the desired effectivity (avL) or quality (PRDN) is reached, the bit flow is stopped. Therefore, SPIHT is suitable for both lossless and lossy compression. The advantage of this method is that the signal is filtered during the compression [46]. In the year 2000, the 1D version of the original 2D SPIHT algorithm was published and applied to ECG [10]. To the best of our knowledge the results outperformed those of all the previously published compression algorithms [6, 10]. SPIHT uses the Temporal Orientation Tree, where one wavelet coefficient in lower frequency bands corresponds to two wavelet coefficients (offspring) in higher-frequency bands or has no offspring. Individual coefficients or the whole trees are coded according to their significance (threshold is used). This algorithm can be used for both physiological and pathological signals. The original related study [10] presents the efficiency CF = 20 and distortion PRDN = 7.52 %. In this work, the algorithm based on WT and SPIHT as described in [11] was used for compression.

### 4.3. Principles of Compressed Signal Evaluation by Experts

Two experts evaluated two leads of their choice (I and V1) of all 125 signals from the CSE database compressed with 33 different values of avL and one without compression (original signal). These two experts are the ones who previously established 4R consensus in [8], where the CSE database was also used. For this purpose, they used an open signal processing software platform SignalPlant [58] and divided the signals into three quality-related groups of their choice according to the diagnostic information preservation. These three groups were as follows:

Perfect quality: evaluable without restrictions (evaluable rhythm, P wave and QRS complex morphology, delineation of all intervals and segments, classification of bundle branch block type, changes of ST segment, and postinfarction changes).

Good quality: some parts of ECG (e.g., P wave, ST segment) are distorted due to compression, and potential of analysis is reduced (evaluable rhythm, QRS complex morphology, delineation of intervals and segments except the P wave, and classification of bundle branch block type).

Nonevaluable ECG: significant distortion or even missing QRS complexes. In this group, only the rhythm could be evaluated (approximately).

For each signal, two boundary values of avL were set (because of the three quality groups). The compressed signal evaluation provided by these two experts was used to create recommendations for the evaluation of ECG signal quality after compression. According to the boundary values of avL, boundary values for each objective method were set.

### 4.4. Evaluation of Compressed Signals Using Different Objective Quality Parameters

The 16 tested quality parameters without diagnostic information are MSE, NMSE, RMS1, PRD, PRDN1, SNR1, SNR2, PSNR, MAX, NMAX, STDERR, CC2, PAD, *κ*, angle, and QS. Not all the metrics described in the review above were tested. Some of the methods produced signals as a result (e.g., LAE, PARE), while some of them were redundant (NRMSE, RMS2), incorrect (CC1), or not useful for our purpose (MAPRD, PRDT). In addition, a comparison between the most used method PRD and three variants of PRDN was carried out. In order to compare the performance of different PRD/PRDN metrics, three variants of the signals were used: (a) with DC component; (b) with DC component and artificially added offset value of 1,024, and (c) with subtraction of DC component (signal without DC and offset). To demonstrate the difference between these algorithms, two values of offset (*P* in (17) and (18)) were also used.

The complete group of methods with diagnostic information based on WT was tested (i.e., PE, WWPRD, WEDD, and MSEWPRD with three variants of weight estimation WSNA, RWSE, and RMWSE). These methods were implemented with the use of two different types of WT: discrete time dyadic wavelet transform with decimation and stationary wavelet transform (SWT).

WPRD is described ambiguously in the existing literature and the source article does not contain any results. This method cannot be applied as it is, because no available algorithm can reliably delineate the Q wave and the beginning of the T wave as well. In fact, in this article, WPRD takes into consideration only P wave and QRS complex distortion. We use three combinations of weights: wP = 0.01, wQRS = 0.99; wP = 0.5, wQRS = 0.5; and wP = 0.99, wQRS = 0.01.

Among CDI, AAE, and WDD, the WDD method is the most complex, taking into account 18 ECG features. As a representative, the WDD was tested. The implementation of the algorithm is very time consuming, dependent as it is on a delineation algorithm and processes of features extraction, because not every detail is included in the existing literature. We used a delineation algorithm [59] and we calculated the features only in beats, where all eight points were delineated. The detection of delta wave (slurred upstroke of the QRS complex; one of the features of the Wolff-Parkinson-White syndrome) is based on the algorithm [60].

For the tested methods: similarity, SE, and +P, annotations of R-wave positions are required. The percentage similarity and HRT methods were also tested. For this purpose, the R-wave detection algorithm [61] was used.

The quality method based on heartbeat classification using multilayer perceptron NN is not suitable in real cases, because the NN should be trained on annotated signals before being tested on similar signals. Partial PRD was calculated using annotations (positions of P onset and T offset), which is not usable in clinical practice, because, in general, the annotations are not available. The fiducial points can be detected using delineation algorithms, while for lower avLs, the algorithms will not be reliable, especially in the case of P wave detection. Therefore, these methods were not tested.

## 5. Results

The performance of various quality measures was compared based on five different criteria: (1) we depicted the average R-D curves for all tested methods; (2) we calculated three features of R-D curves at the important avL limit of 0.8 bps: sensitivity, variability, and sensitivity-variability ratio (SVR); (3) we did the cross-correlation analysis to compare the whole trend of the methods while we also performed cluster analysis to classify the methods in groups according to their trend similarity; (4) we determined the computational demand of the methods; and (5) we also considered the popularity of the methods (Table 1). In each step, unsuitable methods from the specific point of view can be discarded. It is recommended to use a combination of a few of the methods only.

### 5.1. Results of Subjective ECG Quality Evaluation by Experts

Table 2 shows avL boundary values determined by experts to discriminate between three quality groups. The overall avL boundaries can be set as strict (maximum case in Table 2 is a/b bound: avL = 0.80 bps, b/c bound: avL = 0.25 bps). This means that none of the signals with avLs higher than 0.8 bps and 0.25 bps belong to the lower quality group. On the other hand, some signals with lower or equal avL can belong to the better-quality group (e.g., one signal compressed with avL = 0.20 bps can belong to the quality group (a)). The boundaries can also be set as mild (minimum case in Table 2 is a/b bound: avL = 0.15 bps, b/c bound: avL = 0.10 bps), which means that none of the signals with avL ≤ 0.15 bps and avL ≤ 0.10 bps can belong to the better-quality group. Meanwhile, certain signals compressed with, e.g., avL = 0.25 bps, can belong to the quality group (c). In Table 2, the median case is also shown (a/b bound: avL = 0.40 bps, b/c bound: avL = 0.15 bps), which is a compromise between the maximum and minimum cases. We selected avL = 0.80 bps as the most important limit, which separates signals into those with and without any diagnostic distortion. ECG signals compressed with avL ≤ 0.10 bps are absolutely unsuitable for any analysis. Signals compressed with avL > 0.80 bps can be analysed without restrictions (these signals are not distorted in terms of diagnostic information). Each selected boundary value is highlighted in italic in Table 2.

The experts also noticed that ECG signals that contain higher-frequency components (sharper and narrower QRS complexes) can be compressed more efficiently (lower avL). However, signals with left/right bundle branch block need to be compressed more carefully (higher avL). In fact, it is interesting to note that, in pacemaker ECGs, the spike was removed by the compression algorithm with avL ≤ 0.6 bps. When the signal was considered by experts as the change of morphology—which would lead to misdiagnosis—this usually manifests itself as the loss of sharp transitions in ECG components. In such cases, it will not be possible to delineate signals reliably. Following these changes (for lower avLs) the amplitude of signals was also changed. In some signals compressed with an avL in the range of 0.2-0.45 bps, the artefact appears in the segment, where the P wave could be expected (although the P wave was not present in the original signal). These artefacts can be incorrectly considered as a P wave. Lead I was, in general, significantly more distorted by high-frequency noise than lead V1.

### 5.2. Results of Objective ECG Quality Evaluation Using R-D Curves

#### 5.2.1. The Trends of R-D Curves for Different Groups of Methods

The recommended methods for evaluation of the ECG signal quality after compression should be selected according to their performance. For the comparison of ECG signal quality evaluation methods it is advantageous (if not necessary) to take into consideration the results of rate-distortion (R-D) analysis [6]. This is usually performed by an R-D curve (e.g., avL-PRD). In this paper, all the methods were evaluated using the avL method R-D curve. It is always desirable to know the connection between rate and distortion, because increasing lossy compression efficiency is always connected with decreasing signal quality. 

**Figure 1:**
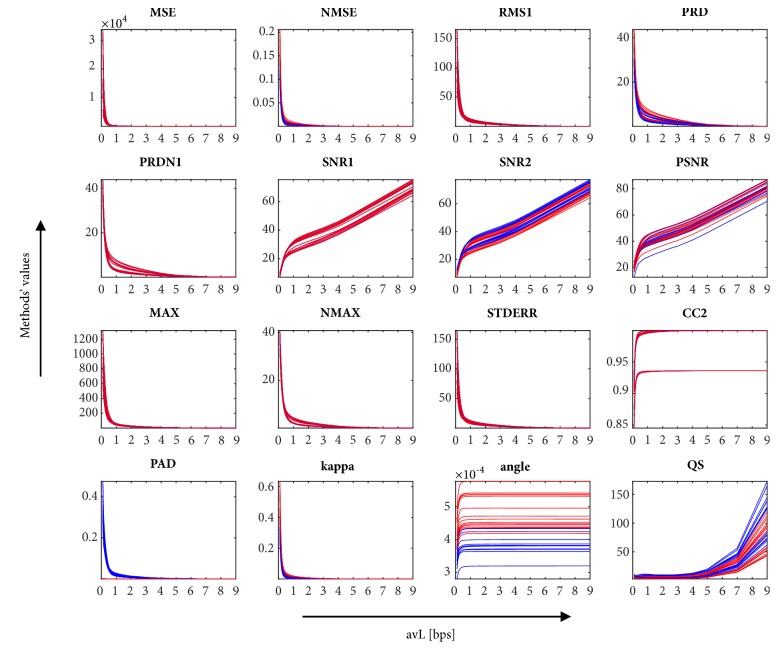
The graphs of the chosen methods (for evaluation of the ECG signal quality after compression) without diagnostic information. On the x-axis there are values of avL in bps; on the y-axis there are values of each method's results. The algorithms were tested on all 15 leads of all 125 signals of the CSE database. The blue and the red curves represent signals where the DC component is present or is subtracted, respectively. One curve represents one averaged lead.

*Methods without Diagnostic Information. *The results of the methods for evaluation of the ECG signal quality after compression that do not include diagnostic information are shown in Figure 1. Here, the graphs show the dependence of the methods' value on avL. The trends of the values of the various methods are, in many cases, very similar; they are hyperbolic or logarithmic. In Figure 1, the influence of subtracting the DC component is illustrated. The blue and red curves represent signals where the DC component is present (blue) or is subtracted (red). In Table 3, the differences in the methods tested on signals with and without a subtracted DC component are shown. The difference values are average differences of each method's values calculated for all 15 leads of all 125 signals and 33 values of avL for the given method. Differences are normalized on the range <0, 1> according to the range of their values on the nonequally sampled interval of avL <0.1, 9>; the average may differ on different avL intervals. The methods that are not sensitive to the subtraction of the DC component are highlighted in italic (STDERR, CC2). The majority of methods are sensitive to the subtraction of the DC component (six slightly highlighted in bold and eight highly highlighted in underline). It should be noted that the difference between PRD and PRDN1 (in Table 3) is two orders. To evaluate the signal quality correctly, we recommend always subtracting the DC component. For other methods described in Section 5.2, the influence of the DC component is not illustrated.

In the case of the DC component subtraction, the PRD and PRDN values are nearly equal and SNR1 and SNR2 are equal, whereas in the case of the DC component presence, these are not equal. For further testing, only SNR1 is used, since we have recommended the extraction of the DC component. If the maximum of the ECG signal is negative, the PSNR is a complex number. Therefore, in this case, the subtraction of the DC component is necessary.

**Figure 2:**
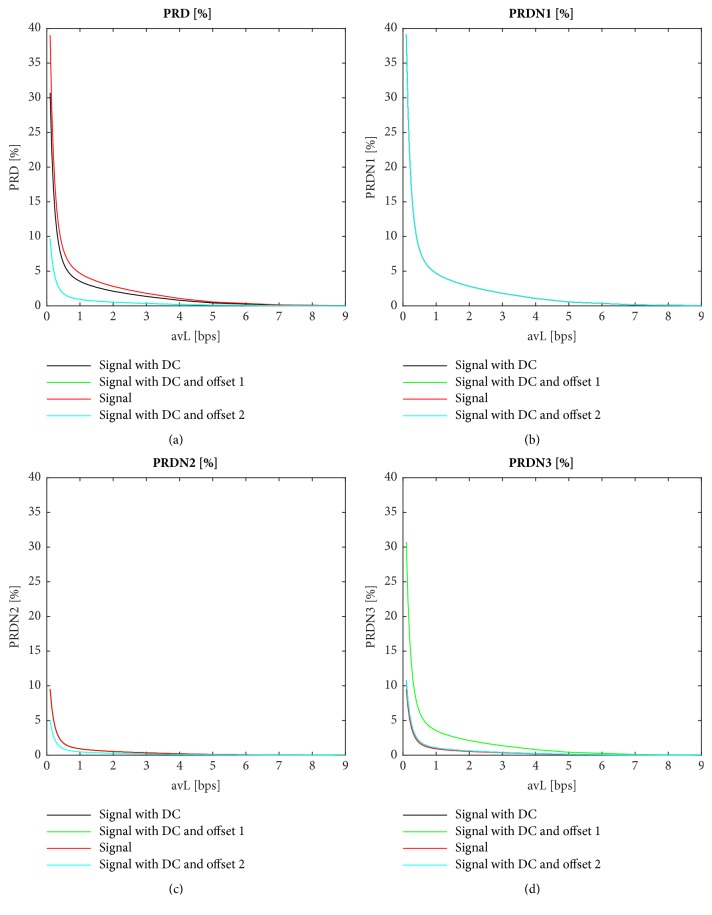
Comparison between PRD and three variants of PRDN. Comparison between PRD and three variants of PRDN tested on signals with DC (black), with DC and offset 1 and 2 (green and cyan, respectively), and with subtracted DC (red). In both types of signals—with DC and offset 1 and 2—the DC component is preserved and offset of 1,024 is added; the difference is in algorithms PRDN2 and PRDN3 where different values of offset P are subtracted (1,024 and 2,048, respectively).

Figure 2 shows a comparison between PRD and three variants of PRDN. It can be seen that PRD produces different results in the case of signals with DC, signals with DC and offsets 1 and 2, and signals without DC and offsets. In both types of signals—with DC and offsets 1 and 2—the DC component is preserved and an offset of 1,024 is added; the difference is in algorithms PRDN2 and PRDN3 where different values of offset* P* are subtracted (1,024 and 2,048, respectively). The lowest PRD is found with a signal where the DC and offset are included. On the other hand, the highest PRD is found in the case of signals without DC and offset. In (16) of PRDN1, the DC component is subtracted and is therefore suitable for signals with or without DC and/or offset. We can say that offset is a particular type of DC. Correctness of this statement demonstrates Figure 2(b), where only one curve for all cases can be seen (curves overlap). The curve is the same as the red curve in Figure 2(a), PRD for the signal, in which the DC component was subtracted. Furthermore, PRDN1 is not dependent on the value of offset. In the case of PRDN2, both offset and DC are subtracted according to (17). However, if these are subtracted as in (17), this is not correct. It is enough to subtract only the DC component in which the offset is included.  Figure 2(c) shows that the resulting curves are not equal for signals with DC and offsets 1 and 2, because in (17), two different values of offset are subtracted. This method is dependent on the subtracted value of the offset. We usually know the correct value, but method PRDN1 is theoretically correct and more comfortable for the user. In the case of (18) of PRDN3, only the offset is subtracted, which is not correct either. The curves in Figure 2(d) then differ and have lower PRDN values than in PRDN1.

Out of these four methods, we recommend that PRDN1 is used, which is universal for signals with or without DC component and offset. Thus, only the PRDN1 method is further analysed.

**Figure 3:**
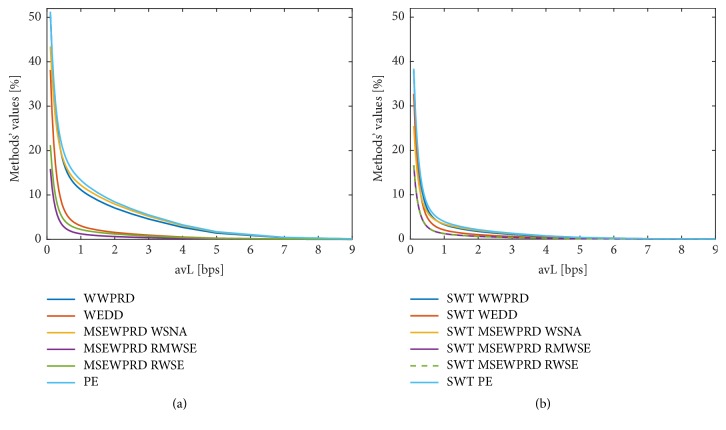
The average curves of methods with diagnostic information based on WT. Two types of WT were tested: (a) discrete time dyadic wavelet transform with decimation; (b) stationary wavelet transform. In the case of SWT, methods MSEWPRD RMWSE (purple line) and MSEWPRD RWSE (green dashed line) have the same trend.

*Methods with Diagnostic Information Based on WT. *As shown in the graphs in Figure 3, all six indexes based on WT have similar trends. Two types of WT were tested: discrete time dyadic wavelet transform with decimation (Figure 3(a)) and stationary wavelet transform (Figure 3(b)). In the case of SWT, the range of values is lower and two methods, MSEWPRD RMWSE and MSEWPRD RWSE, have equal trends. With the increasing effectivity of compression (decreasing avL), the indexes (distortion) increase as well. The results of these methods depend on whether the DC component is subtracted or not, which is not illustrated for these methods. In Figure 3, only the average curves calculated from all signals and leads are shown.

In Table 4, there are five quality groups, as described in Section 3.2.2, and their boundary values for PRD [3], WWPRD [3], and WEDD [28] as well as the correct prediction value CP. Based on the CP, the WEDD method is considered as the most appropriate. The new factor in this table are values of avL (highlighted in italic) determined from our graphs (one average R-D curve for each method calculated from all 125 signals and 15 leads; the DC component was subtracted). In all cases, the avL whose value corresponds with the nearest smaller value of PRD, WWPRD, and WEDD was picked. CP informs us on the percentage of signals that were classified correctly into the quality group [3, 28]. In Table 4 it can be seen that only the avL_WEDD_ (0.7 bps) determined on the basis of WEDD values for excellent quality group has similar value to our boundary (avL = 0.8 bps set by two experts) between perfect and good signals. Therefore, the boundaries of PRD and WWPRD should be set from our R-D curves at a limit avL of 0.8 bps (see Table 5). 

**Figure 4:**
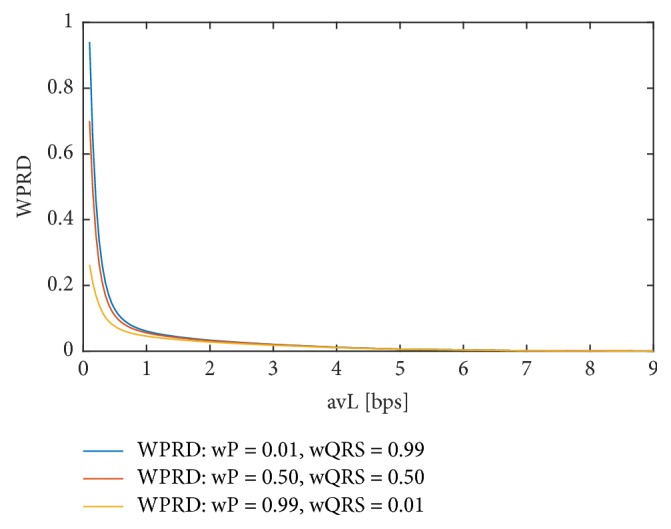
Values of WPRD tested with different settings of weights for P wave and QRS complex.

*Methods with Diagnostic Information Based on Delineation. *In Figure 4, the results of WPRD method are shown. The curves were calculated from WPRD values for all signals and leads. The blue curve represents the result of WPRD with higher weights for QRS complex (wQRS = 0.99 and wP = 0.01), while with the red curve, the weights for P wave, and QRS complex were set equally (wP = 0.50, wQRS = 0.50). Meanwhile, the yellow curve shows the results of WPRD with higher weights for P wave (wP = 0.99, wQRS = 0.01). The results of this method are dependent on the settings of weights. From the curves it follows that the QRS complex is more distorted than the P wave in signals compressed with lower avL (approx. 0.1-0.4 bps).

It is very difficult to extract features from ECG signals without preprocessing (e.g., baseline wander elimination). The results of WDD are highly dependent on using a delineation algorithm, the feature extraction method, and signal character. On the other hand, the signal quality after compression is evaluated in relative terms (the use of the same delineation algorithm and the same means of feature extraction for both the original and compressed signal). This index is very complex and also very tricky. There exists no perfect delineation algorithm or any perfect setup of the WDD algorithm that can be used universally for any signal. The WDD algorithm is very computationally demanding. It is also very difficult to detect delta waves using delineation algorithms based on WT. The delta wave is of low frequencies and this means that WT-based delineation algorithms do not include it correctly in the same band as the QRS complex, which is of higher frequencies. WDD value was calculated using only those ECG beats where all eight fiducial points were detected in both original and reconstructed signal (this generally means that the WDD for signals with lower avL is calculated on a smaller number of beats than with signals with higher avL).

**Figure 5:**
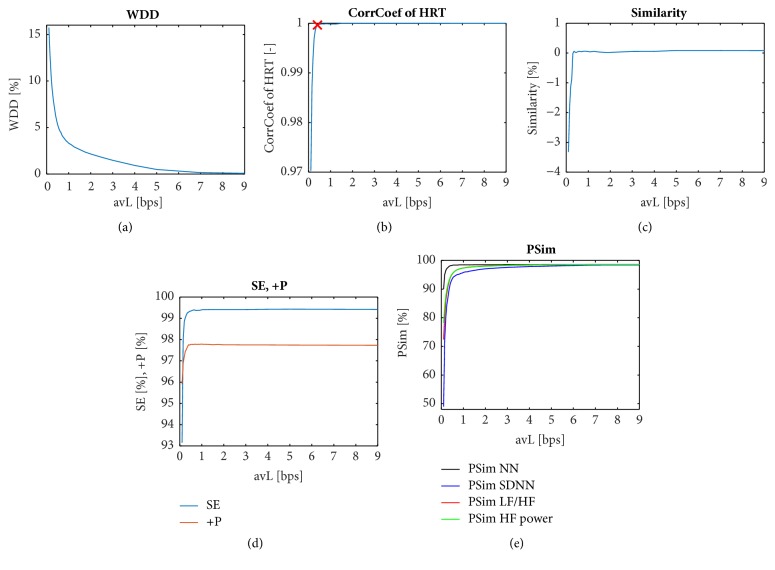
The resulting curves of the nine methods. (a) WDD, (b) CorrCoef of HRT, (c) similarity, and (d) sensitivity (SE) and positive predictivity (+P), and (e) PSim NN, PSim SDNN, PSim LF/HF, and PSim HF power were calculated as a mean of each of the 15 leads of all 125 ECG signals. The trends of the methods CorrCoef of HRT, SE, +P, and all four PSim methods are similar, although each method has a different range of values. The WDD method has a trend similar to the majority of the previously mentioned methods. Similarity has nonmonotonous trend and also incorrect values. Therefore, it cannot be recommended for further use. The red cross in (b) marks the breaking point in the quality of the signal. With upward trend of the value of avL, the CorrCoef of HRT changes only slightly, while with downward trend of the avL value, the CorrCoef of HRT sharply decreases.

The resulting graph of WDD is shown in Figure 5(a). As can be seen, the trend of WDD is hyperbolic and very similar to WPRD and is also similar to all the methods based on WT and some of the methods without diagnostic information.

The original ECG signals and the signals compressed with different avLs were compared in terms of HRT using a correlation coefficient (CorrCoef), which is pictured in Figure 5(b). The correlation is very high—more than 0.97 even in the case of the lowest avL—and it rises up to one for the highest values of avL. As for the breaking point in the quality of the signal, we can consider this to be the one where avL = 0.40 bps (red cross in Figure 5(b)). From this value of avL upwards (to the right in Figure 5(b)), the quality (CorrCoef of HRT) changes only slightly. The advantage of this method lies in the use of a QRS detector instead of a complex delineation algorithm. The use of the QRS detector is more accurate and less computationally demanding. On the other hand, when using this algorithm, only rhythm is considered.

Similarity, as described in [26], is according to (43) rather dissimilarity, because the smaller the difference between the detected and annotated values is, the smaller the similarity is. There is no absolute value in the equation and therefore, similarity also has negative values (see Figure 5(c)). Moreover, this method has nonmonotonous trend. This method definitely cannot be recommended for further use and will not be further analysed.

The values of sensitivity and positive predictivity of QRS complex detection for all signals from the CSE database are shown in Figure 5(d). Sensitivity ranges between 93 % and 100 %. Positive predictivity has an even narrower range of between 95 % and 98 %. The values of SE and +P sharply increase from the lowest avL of 0.1 bps to the avL of 0.4 bps while for higher avLs, the values are almost constant. This is not advantageous, because we need the sharpest increase in the area of around avL = 0.8 bps.

The results of the PSim method are shown in Figure 5(e), where different features (NN, SDNN, LF/HF, and HF power) are pictured. These features have a very similar trend.

#### 5.2.2. Sensitivity and Variability Analysis of R-D Curves Trends

**Figure 6:**
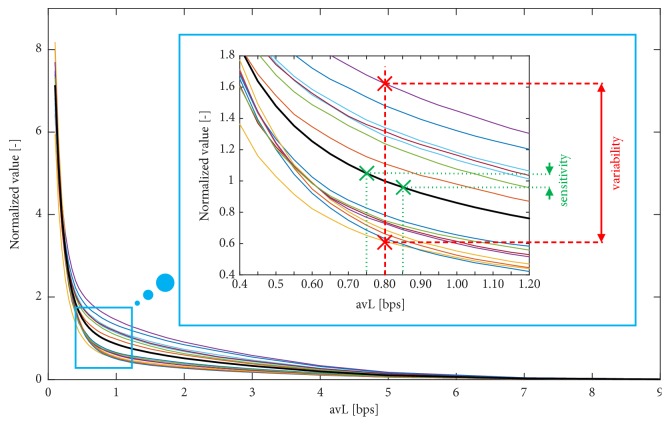
Illustration for calculation of sensitivity and variability (standard deviation in a statistical sense of meaning). Normalized curves for all 15 leads are the thin colour lines; average curve calculated from the 15 curves is the thick black line. The blue frame zooms on an important part of the graph for the area around the important avL limit of 0.8 bps. In this frame, the calculation of sensitivity and variability is illustrated. Sensitivity (highlighted in green) is calculated from the average curve as an absolute value of a difference of its values at avL = 0.75 bps and avL = 0.85 bps (marked by green crosses). Sensitivity is an approximation of the derivative of the curve at avL = 0.8 bps (approximation of the slope of the tangent line to the curve at that point). The area from which the variability is calculated is defined by the dashed red line delimited by two red crosses. From the points of all 15 curves at avL = 0.8 bps (defined area), the standard deviation in a statistical sense of the meaning is calculated.

From the R-D curves, the sensitivity, variability, and sensitivity-variability ratio (SVR) properties are calculated. These features are computed for the most important limit of avL = 0.8 bps (the selection of this value is described in Section 5.1 in detail). The procedure of calculation of these parameters (supplemented by Figure 6) is as follows. First, the average curve from all 15 leads is calculated. Then, the average curve as well as all 15 curves for all 15 leads are normalized, divided by the value of average curve at avL = 0.8 bps (colour curves in Figure 6, the average curve is thick black). The sensitivity is calculated from the average curve as an absolute value of a difference of its values at avL = 0.75 bps and avL = 0.85 bps (neighbouring values of the limit avL = 0.8 bps). This is an approximation of the derivative of the curve at avL = 0.8 bps (approximation of the slope of the tangent line to the curve at that point). The higher the sensitivity is, the better it is, because such a method can greatly distinguish between signals with and without any diagnostic distortion. Variability is a standard deviation in a statistical sense of the meaning; it is calculated from points of all 15 curves at avL = 0.8 bps. The lower the variability is, the better it is. This means that the method has similar results for all ECG leads (i.e., it is more universal). Sometimes it can be difficult to assess the quality of the method using these two features; we therefore created their combination and called it SVR (ratio between sensitivity and variability). The higher the value of the SVR is, the better the method is for signal quality assessment.

**Figure 7:**
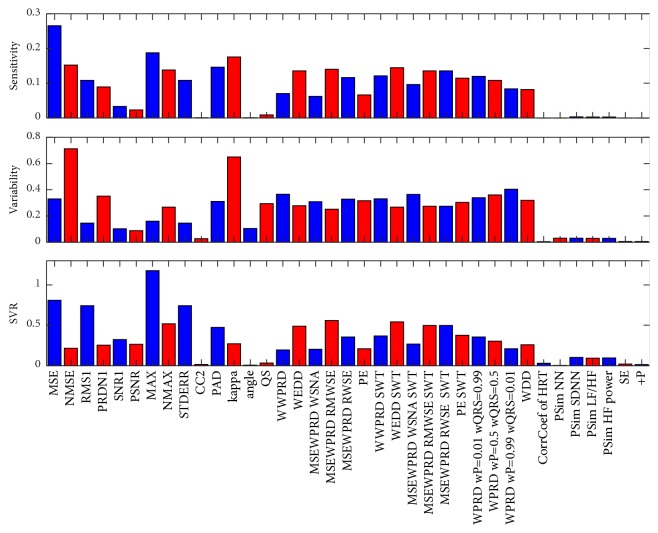
The bar graph of sensitivity, variability, and SVR results. For better readability, neighbouring bars are separated by two different colours, blue and red. Some bars are of very low value; therefore they are not visible. If the sensitivity is high and variability is low, then the SVR is high and the method can be recommended for further use from this point of view.

Figure 7 shows the results of sensitivity, variability, and SVR in a form of a bar graph. For better readability, neighbouring bars are separated by two different colours: blue and red. Some bars are not clearly visible, because the values of sensitivity, variability, and SVR are very low for some methods for the assessment of ECG quality after compression.

A WPRD with three different settings of weights has different sensitivity, variability, and SVR. Thus, it is obvious that weights influence these three features. For further analysis, as an example, only the WPRD with equal weights for P wave and QRS complex will be used since potentially, there is an infinite number of weights settings.

Methods based on SWT have better results of WWPRD, WEDD, MSEWPRD RWSE, and PE (in terms of higher sensitivity, lower variability, and higher SVR). MSEWPRD WSNA has higher values of sensitivity and SVR, but also a higher value of variability. MSEWPRD RMWSE method has lower values of sensitivity and SVR and a higher value of variability when using SWT than when using WT with decimation.

Methods with low values of SVR (under an empirically set threshold of 0.1), CC2, angle, QS, CorrCoef of HRT, PSim NN, PSim LF/HF, PSim HF power, SE, and +P, are valid in principle and can be used for signal quality assessment, while they cannot be recommended as the best methods.

The highest values of SVR have MAX, MSE, RMS1, and STDERR.

RMS1 and STDERR have almost the same values of sensitivity, variability, and SVR (the highest difference is 3.3307×10^−16^ in the case of SVR).

#### 5.2.3. Cross-Correlation Analysis of R-D Curves Trends

**Figure 8:**
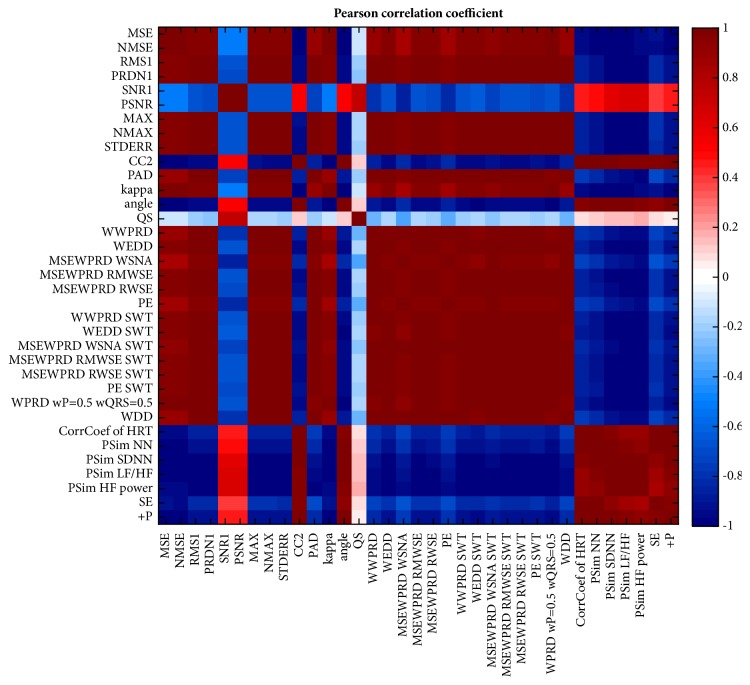
Colour map of Pearson's correlation coefficients for 35 quality methods. Red represents positive correlation, blue negative correlation and white zero correlation.

To compare the specific trends of individual quality evaluation methods results, we carried out cross-correlation analysis. For this, we used Pearson's correlation coefficient. The results are shown in Figure 8. Positive correlations of trends are coloured in red, while the negative correlations are coloured in blue and zero correlation is represented by the white colour. From Figure 8 it can be seen that the trends of the majority of the methods are highly correlated (in a positive as well as in a negative way). The SNR1 and PSNR methods are less correlated with other methods. The only method that is not correlated with any other is the QS. From these facts it follows that we can divide the methods into groups.

**Figure 9:**
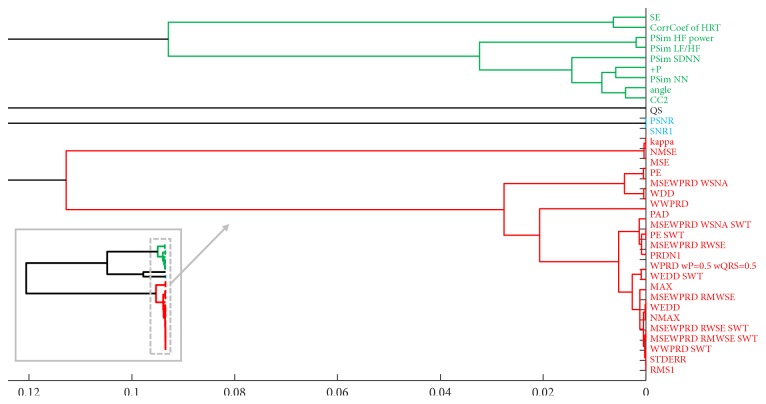
Dendrogram for 35 methods for compressed ECG signal quality assessment. In the left lower corner the whole dendrogram is shown; the dashed rectangular highlights the details of the larger version depicted. The resulting four groups are highlighted in green, black, blue, and red.

The division is performed using cluster analysis in a form of an agglomerative hierarchical cluster tree with a correlation distance measure metric. As a method of clustering, we set a weighted pair-group method using arithmetic averages (WPGMA), because we expect that the resulting clusters will not be equally sized. The constructed dendrogram is shown in Figure 9.

In Figure 9, there is a large-detail dendrogram as well as a complete version (in the left lower corner). Setting the threshold value of the dendrogram at 0.12, the methods were separated into four groups according to their trends. The threshold was set according to the trends of the methods and according to the amalgamation schedule (linkage distance). The resulting groups are highlighted in green, black, blue, and red. The two big groups (green and red) are negatively correlated and their trends are visually different; they are symmetric with respect to the x-axis. This means that the values of the methods in the red group decrease with an increasing avL (decreasing effectivity of compression); these methods can be called error or difference methods. On the other hand, the methods from the green group have higher values for higher avLs and these methods can be called similarity or quality methods. The black group contains only one method, QS, which has a diverse trend not correlated with any other method. The last group—the blue one—contains two methods, SNR1, and PSNR.

RMS1 and STDERR have zero linkage distance. Therefore, we can consider these methods as equal.

From the results of the cross-correlation analysis and from the subsequent dendrogram it follows that we can generally divide the methods into four groups based on their trends.

### 5.3. Recommendations for Method Usage

In Table 5, there are recommended limits of distortion for the methods that were not discarded in the previous sections (i.e., the methods suitable for signal quality assessment). Two more methods, SE and +P, are not shown in Table 5, because they need reference positions of the QRS complex (annotations), which are not at our disposal in practice. WPRD is not shown in Table 5 either, because it has many possible settings of weights and these should be set by cardiologists. Thus we cannot recommend this method. The recommendations are determined in three ranges according to Table 2. The ranges are more or less strict. Particular values in Table 5 were determined from the average curve (all 15 leads, all 125 signals) of each method at the limit avLs. The DC component was subtracted in all the methods except for HRT and PSim (4 variants). If the compression is performed with better (herein better means lower or higher in dependence of method) quality/difference value than stated in the first column of selected case (either min, max, or median), the signal is of “perfect quality”: evaluable without restrictions (rhythm, P wave and QRS complex morphology, delineation of all intervals and segments, classification of bundle branch block type, changes of ST segment, and postinfarction changes). If the value is in the range determined by boundary values in both columns of selected case, the signal is of “good quality”: some parts of ECG (such as P wave, and ST segment) are distorted due to compression'. Here, the analysis potential is reduced (rhythm, QRS complex morphology, delineation of intervals and segments except the P wave, and classification of bundle branch block type). Finally, if the value is even worse than the one in the second column of the selected case, the signal is “nonevaluable ECG”: significantly distorted or has a missing QRS complex. In this group, only the rhythm can be approximately evaluated. Each method in Table 5 is also classified into one of three groups, which inform us about the computational demand of the method (1: low, 2: medium, and 3: high).

All of the methods in Table 5 are valid and can be used for the assessment of signal quality after compression. A combination of all of these methods can make the assessment of signal quality very complex but can result in high computational demand and is very time consuming. Nevertheless, we recommend the use of more than one method for any evaluation of ECG signal quality after compression. We took into consideration the high diversity of the combination of recommended methods. The ideal option seems to be a combination of methods from each group: without diagnostic information, with diagnostic information based on WT, and with diagnostic information based on delineation and also from each cluster (to combine methods with various trends). Thus, we selected the eight most suitable methods. The selection was based on five criteria: (1) the trend of the R-D curves; (2) the results of sensitivity, variability, and SVR at avL = 0.8 bps; (3) cross-correlation analysis and its dendrogram; (4) principle and computational demand; and (5) popularity of the methods (Table 1).

From the first cluster, we selected only PSim SDNN, which has the highest SVR of this cluster. The PSim SDNN method is based on QRS complex detection, so we can expect higher robustness and less computational demand than with methods based on full delineation. On the other hand, CC2, angle, CorrCoef of HRT, PSim NN, PSim LF/HF, and PSim HF power have low SVR and are therefore not recommended.

Although QS has very low SVR, it is the only representative of the second cluster since it has a different trend than any other method, and we recommend it. Its advantage (in terms of diversity of methods) lies in the fact that it combines quality measure with efficiency measure.

The third cluster has two representatives, SNR1 and PSNR. These two methods are highly correlated, and therefore only one of them may be used. We recommend the use of SNR1, because it has higher SVR than PSNR and it is less sensitive to the subtraction of DC than PSNR. It is also the third most cited method (Table 1).

The biggest group is the fourth cluster, from which we recommend five methods, MSE, PRDN1, MAX, STDERR, and WEDD SWT. Here, MSE has the second highest SVR, has low computational demand, is only slightly sensitive to the subtraction of DC, and is the fifth most cited method. PRDN1 was selected because it is the most cited method (Table 1). Furthermore, PRDN1 is universal for signals with or without DC component and offset (instead of PRD). MAX has the highest value of SVR (the second highest sensitivity and very low variability), is the sixth most cited method, and informs us about the highest absolute distortion of the compressed signal. On the other hand, its value is dependent on the signal magnitude (it is not normalized). STDERR is not sensitive to the subtraction of DC, is the tenth most cited method, and has high SVR. This method has almost the same results as RMS1 in the case of signals with subtracted DC. Therefore, we can consider RMS1 as redundant. Methods with diagnostic information based on WT seem to be very good for signal quality evaluation. They contain information about diagnostic quality and they do not need any delineation algorithm. On the other hand, their computational demand is higher due to the WT but is still lower than with the methods based on delineation. From the methods based on WT, we recommend WEDD SWT, because it is the most cited method of WT-based methods and the eighth most cited method of all. It also has the second highest SVR among the WT-based methods. The most complex method from the fourth cluster—WDD—cannot be recommended. Although it includes diagnostic information and is the second most cited method (Table 1), it is extremely computationally demanding and is also dependent on the robustness of the delineation algorithm (more details are in discussion).

Using a combination of methods, we can see the quality of the signal from various points of view (possibilities of their combination is discussed in Section 6). Our own selection of methods is only one possible combination and was selected on the basis of five criteria. This article offers enough information to enable any user to select his or her own combination of methods, which will be suitable for his or her specific purposes.

Methods with diagnostic information using delineation algorithms have great potential because they inform us about the distortion in various parts of ECG. However, their big disadvantage is the necessity of using a delineation algorithm, and a perfect and universal version for ECG signals does not exist. Thus, these quality assessment algorithms are less robust than others.

The recommendations are 100 % valid for the CSE database and compression algorithm based on WT and SPIHT. Each compression algorithm is based on different principle and has varying extents of different properties. They can, for example, simultaneously filter noise (much like SPIHT) or change the amplitude of QRS complexes. With this in mind, it is not possible to determine one-hundred percent, generally valid recommendations for the evaluation of ECG signal quality after compression and reconstruction (valid for any possible ECG signal and any possible compression algorithm). On the other hand, the principle of any ECG signal quality evaluation after any type of compression remains always the same.

## 6. Discussion

It is always necessary to decide for what purpose the signal after compression will be used. As was noted from the outset, the compression is a compromise between the amount of data and their quality. In addition, the reflection of diagnostic distortion is an important parameter of the methods as well as their relative computational demands. From the cross-correlation analysis, it follows that many methods—from the simple ones to those based on delineation—have highly correlated trends. Furthermore, aside from trend and cross-correlation, the sensitivity, variability, and SVR of the methods are important features in terms of the relative suitability of the methods assessed. For example, MAX—a method without diagnostic information that has similar trends to WEDD SWT (they are from the same cluster) and is less computationally demanding—has higher sensitivity and SVR and lower variability. Therefore, it seems that it may be sufficient to use only computationally less demanding methods. Nevertheless, our recommendation is to use a combination of diverse methods to assess the signal quality after compression. This way, we can look at the signal with more complexity. Moreover, each method can fail in some specific cases, e.g., MAX in the case of impulse noise presence. Therefore, this new compression scheme—the combination of methods—enables more robust quality assessment.

MAX error has the highest value of SVR and although it seems very simple, it was selected into those eight recommended methods. It informs about the maximum difference in signal amplitude. If it is low enough, the signal cannot be misdiagnosed (e.g., if the value of MAX is approximately equal to quantization step size, it means that the distortion of the reconstructed signal comes up to the level of quantization noise). Moreover, it is only one method from eight selected methods; it is only one of eight points of view, which brings us valuable information about distortion of the signal.

One of the possible combinations of the eight recommended methods in practice could be as follows. First all eight methods are applied on a pair of original and compressed signals. According to Table 5 the quality group for each signal and method can be determined. Finally, if the signal belongs to the highest quality group for all eight methods, this signal is considered as of perfect quality and can be further used. This is the strict method. Another possibility is more moderate. Here, the signal can be considered of perfect quality if values of at least five of the methods exceed the higher threshold of the method (the column with higher avL in Table 5). In addition, the thresholds for the minimum, maximum, and median cases can be considered. Thus, we have many possible combinations, while these are definitely not exhaustive. In fact, the combination of methods for the assessment of signal quality after compression can be set up according to the user's specific needs.

To evaluate the signal quality correctly, we recommend always subtracting the DC component. This way, we can avoid artificial low or high values of quality indexes and we do not have to know whether the method is or is not sensitive to the subtraction of the DC.

If we consider the content of diagnostic information alone as the most important criteria for the selection of the best quality index, the result of this selection will be the WDD method. This method is the most comprehensive one and has the highest potential. This method—along with other methods described in Section 3.2.3—requires the use of a delineation algorithm, a flawless version of which does not exist. These algorithms can thus introduce an artificial error into ECG quality assessment. This is an essential problem especially in case of pathological signals, where the delineation algorithms most often fail. We can say that WDD is a timeless method, it will probably give reliable results when it will be used with flawless delineation algorithm. Moreover, these methods are extremely computationally demanding and time consuming and cannot be applied online, which can be problem, for example, in telemedicine. In fact, this may also be a problem in the methods based on WT.

We also briefly mentioned single-lead ECG quality assessment methods, which are not directly connected with compression but they can be probably used in this area. The disadvantage of these methods is the fact that they assess the quality of compressed signal only. The other methods use two signals, original and compressed ones to evaluate the quality of the compressed signal. Using the single-lead methods we would lose this information. Therefore, the methods are not described in detail in this article.

In this work we used avL as a method for evaluation of compression efficiency. It has clear units and we can compare its value with the bit resolution of the original signal. In literature, also other methods such as CF and CR are frequently used. CF and CR can be calculated using avL. avL is not directly influenced by sampling frequency such as CDR. However, it is influenced indirectly. The value of numerator of the avL equation (number of bits after compression) is dependent on the compression algorithm (its principal and specific setting). If we upsample the signal, e.g., twice, the signal will be twice longer and it will be represented by twice higher number of bits. However, each compression algorithm can compress the original and the upsampled signal differently. Therefore, the output signal will be expressed by different number of bits (it will not be exactly twice higher than the original one).

To the best of our knowledge, our method of subjective quality assessment (reference) is different from any previously published method in the area of compression. The two experts on ECG signals designed the subjective method of ECG signals quality evaluation after compression and thus determined the reference. They preferred this method over MOS, because it is less time consuming and it is approximately as detailed as MOS in terms of evaluated diagnostic information. Our experts evaluated the signals in the same way as they do it in daily practice. Of course, it is not the only possible method, but we consider it as a reasonable and logical alternative. On the other hand, if, e.g., MOS method is used, the results can be compared with some other authors. MOS can assume values from 0 % to 100 %; thus the correlation between MOS value and the objective methods' values can be accomplished. Signals in our method are classified into one of three diagnostic groups, which is sufficient from the medical point of view. In [35], the authors use MOS and four compression algorithms, but they do not consider various values of compression efficiency of each algorithm; they classified the signals into four quality groups. In [3], the authors used MOS methods as well, but they use different four algorithms as well as signals than in [35]; they classified the signals into five groups. Both articles tested the PRD measure and reach different results. Even in the case of known subjective MOS measure, the methodology and results are not standardized. We consider only one compression method but 33 values of compression efficiency (avLs) from which the avL = 0.8 bps was determined by the experts as the most important one. If the signal is compressed using avL higher than 0.8 bps, then the diagnostic information was not distorted in any case; the experts evaluated all compressed signals the same as original signals (not compressed). We worked with the whole CSE database and our experts evaluated two leads of all signals compressed with 33 different avLs; altogether it was 8,250 signals. It will be extremely time consuming to assess this amount of signals using MOS method. Taking into consideration various efficiency of the compression algorithm, we can plot the R-D curves of the methods and perform the cross-correlation and cluster analysis.

Here, we compare previously published recommendations, which were mentioned in Section 3 and Table 4 of this paper with our new recommendations. The authors of [13, 35, 42] stated that signals compressed with PRDN < 9 % are of very good or good quality; this result was obtained for four tested compression algorithms. Our most strict recommendation sets the limit of PRDN = 5.4 %. In [7] the authors recommend compressing with avL ≥ 0.8 bps with a corresponding value of PRDN $\dot{=}$ 5 % using a WT and SPIHT algorithm. The algorithm was tested on 12 standard leads of the CSE database. In our study, the value of PRDN = 5.4 % in the case of avL = 0.8 bps. In [39] the authors recommend compressing with WWPRD < 10 % using WT and SPIHT algorithm and tested it on the whole MIT-BIH Arrhythmia Database and MIT-BIH Compression Test Database. Our strictest recommendation determined the limit at 12.6 %. In [4] the authors set the borders of WEDD at 2 % and 5 % using the WT and SPIHT compression algorithm and short segments of some signals from the MIT-BIH Arrhythmia Database. If the signal has WEDD ≤ 2 %, it is of very good quality, while if it has WEDD ≥ 5 % it is of very bad quality [4]. If the signal has WEDD between the boundary values, it is of intermediate quality. Our strict boundary values of WEDD (based on discrete time dyadic wavelet transform with downsampling) are 3.8 % for the perfect quality and 17.0 % and above for nonevaluable signals.

According to this comparison between the recommendations of previously published papers and our test results, it seems that the boundary values of any method for evaluation of the ECG signal quality after compression depend on the compression algorithm used as well as the signal database that was used for testing. Therefore, the recommendations are one-hundred percent valid in the cases where the same algorithm and database are used, while they are valid only in terms of “a rough guess” in the cases where different algorithms and/or databases are used. Generally, it is not possible to determine boundaries of quality evaluation methods, which will be 100 % valid for any compression algorithm and any signal. It is not within the compass of any author and any paper. Suffice it to say, the methodology that was used in this review for providing the recommendations is valid in any specific case.

### 6.1. Considerations on Compression and Noise

Almost every ECG signal contains some type(s) of noise (e.g., myopotentials, baseline fluctuations = drift, and 50 Hz power line interference). While filtration usually does not precede compression, some compression algorithms such as those based on WT filter noise (it is a positive side effect of compression). This phenomenon is desirable, while if the compressed and simultaneously filtered signal is compared with the original noisy signal, this will negatively manifest itself in the value of the majority of the methods for evaluation of the ECG signal quality after compression. The values of the quality indexes are artificially higher (in the case of distortion methods) or lower (in the case of similarity methods). For example, an ECG signal with higher PRD (caused artificially by noise reduction) can be of more quality and be more valuable from a diagnostical point of view than an ECG signal with lower PRD (without filtration ability). Two quality assessment methods somehow consider noise, as their authors noted: WEDD [46] and MSEWPRD [31]. Here, there is the possibility of comparing the compressed and filtered signal with the original signal without noise. However, this solution is not simple either. The noiseless original signal can be obtained by using a filtration algorithm that can cause changes in diagnostic quality. The filtering process is also computationally demanding.

## 7. Conclusion

Lossy compression is always a compromise between the amount of data and their relative quality. Therefore, the assessment of ECG signal quality after compression and the determination of compression efficiency should be an essential part of compression itself. The authors of previously published papers use various algorithms for the ECG signal quality assessment. However, there exists neither a standard nor a unified approach. The most popular method is PRD, which belongs to the group of objective methods without diagnostic information. This method has a few variants, which are not always used properly and can thus result in artificially lower PRD. Therefore, we recommend the use of a PRDN1 variant (among other methods). To evaluate the signal quality correctly, we recommend always subtracting the DC component. It is also advantageous to calculate and figure the R-D curve. In terms of an efficiency measure, we used avL because of its clear definition and its facilitation of making comparisons with the bit resolution of original signals. The suitability of the methods for quality assessment was evaluated based on five criteria: (1) the trends of the R-D curves; (2) the results of sensitivity, variability, and SVR at avL = 0.8 bps; (3) cross-correlation analysis and dendrogram; (4) principle and computational demand; and (5) popularity of the methods (number of times cited). For the assessment of ECG signal quality after compression we recommend using a combination of selected methods. This enables complex views at the signal, because of the diversity of the methods (they are based on different principles and their R-D curves are of four different trends). The recommended methods are PSim SDNN, QS, SNR1, MSE, PRDN1, MAX, STDERR, and WEDD SWT. All of these methods are low or medium in terms of computational demand and do not need a delineation algorithm, which can introduce an artificial error into ECG quality assessment. Any user can combine the methods in a different way and this review offers enough information to enable this. According to the experts' assessments, two thresholds of signal quality were determined for each method. The thresholds separate out three signal quality groups, which leads to appropriate further analysis. According to the concrete thresholds, the user will know whether the compressed signal is of sufficient quality for his or her specific purpose.

## Figures and Tables

**Table 1:** Popularity of methods for evaluation of ECG signal quality according to Scopus. In columns 3-5 there is also ticked group to which each method belongs.

Method	No. of articles	Without diagnostic information	Based on WT	Based on delineation
PRD, PRMSD, MPRD, PRDN	273	✓		
WDD	126			✓
SNR, PSNR	93	✓		
RMS, RMSE, NRMSE	78	✓		
MSE, NMSE	60	✓		
MAX, MAE, PE, MaxErr, NMAE, NMAX	55	✓		
CC, NCC	27	✓		
WEDD	23		✓	
QS	21	✓		
STDERR	19	✓		

**Table 2:** Boundary values of avL for classification of compressed ECG signals (leads I and V1) into 3 quality groups (a: perfect quality, b: good quality, and c: not evaluable ECG). The values come from two experts (1 and 2). The overall avL boundaries can be set as strict-max case, mild-min case, and medium-median case.

	lead V1: bound a/b		lead V1: bound b/c		lead I: bound a/b		lead I: bound b/c	
	expert 1	expert 2	expert 1	expert 2	expert 1	expert 2	expert 1	expert 2
min avL	0.20	0.20	*0.10*	0.10	*0.15*	0.20	0.10	0.10
max avL	0.70	*0.80*	0.20	*0.25*	0.65	0.65	0.20	0.20
median avL	*0.40*	0.35	0.10	*0.15*	0.30	0.30	0.10	0.15

**Table 3:** Differences of methods tested on signals without and with subtraction of the DC component. The methods that are not sensitive to subtraction of the DC component are highlighted in italic, and slightly sensitive methods are highlighted in bold, while highly sensitive methods are highlighted in underline. The difference values are average differences for all samples of all curves for the given method. Differences are normalized on the range <0, 1> according to the range of their values on the not equally sampled interval of avL <0.1, 9>; the average may differ on different avL interval.

MSE	**0.000520**	MAX	**0.000575**
NMSE	0.029893	NMAX	**0.000630**
RMS1	**0.000488**	STDERR	*1.25E-17*
PRD	0.046018	CC2	*5.06E-15*
PRDN1	**0.000447**	PAD	0.147074
SNR1	**0.000213**	kappa	0.032468
SNR2	0.042550	angle	0.625083
PSNR	0.014173	QS	0.033148

**Table 4:** Signal quality groups separated by values of PRD [3], WWPRD [3], and WEDD [28] with corresponding values of avL determined using the mean of index values for all signals and leads of the CSE database.

quality	PRD	CP	avL_PRD_	WWPRD	CP	avL_WWPRD_	WEDD	CP	avL_WEDD_
group	[%]	[%]	[bps]	[%]	[%]	[bps]	[%]	[%]	[bps]
excellent	0-4.33	100	*1.15-9*	0-7.4	100	*2-9*	0-4.517	100	*0.7-9*
very good	4.33-7.8	60	*0.55-1.15*	7.4-15.45	96	*0.6-2*	4.517-6.914	98	*0.5-0.7*
good	7.8-11.59	36	*0.4-0.55*	15.45-25.18	93	*0.35-0.6*	6.914-11.125	93	*0.35-0.5*
not bad	11.59-22.57	20	*0.2-0.4*	25.18-37.4	82	*0.2-0.35*	11.125-13.56	91	*0.3-0.35*
bad	> 22.57	27	*< 0.2*	> 37.4	89	*< 0.2*	> 13.56	93	*< 0.3*

**Table 5:** Recommendations for compression. All the methods suitable for assessment of signal quality after compression are listed. These methods are separated into four groups according to the cluster analysis. Each group is separated by a horizontal line. The recommendations are determined in three ranges (min, max, and median case) according to Table 2. Using this table, the signals after compression can be divided into three groups of quality and the user does not have to know or set avL. The last column of this table informs us about the computational demand of the methods (1: low, 2: medium, 3: high). On the basis of five criteria, we recommend a total of eight methods for signal quality evaluation, which are highlighted in bold. The methods are diversified, which means that they come from all four clusters (which represent four different trends) and are based on various principles. The other methods from the table can be used as well since they are valid, while they are not the best.

	the min case		the max case		the median case		computational demand
	avL = 0.15	avL = 0.1	avL = 0.8	avL = 0.25	avL = 0.4	avL = 0.15	
CC2	0.9471	0.9078	0.9851	0.9728	0.9816	0.9471	1
angle	0.0004	0.0004	0.0005	0.0005	0.0005	0.0004	1
CorrCoef of HRT	0.9674	0.8872	0.9975	0.9861	0.9963	0.9674	2
PSim NN	95.0655	89.9840	98.4245	97.1784	98.1705	95.0655	2
**PSim SDNN**	**72.2802**	**49.0112**	**95.3552**	**84.4947**	**91.8483**	**72.2802**	**2**
PSim LF/HF	80.7772	72.3930	96.9980	88.6365	93.9449	80.7772	2
PSim HF power	84.0985	78.1634	96.9640	89.9535	94.0963	84.0985	2

**QS**	**4.7743**	**5.3715**	**5.5365**	**4.7622**	**5.1795**	**4.7743**	**1**

**SNR1**	**11.5943**	**9.0804**	**26.6386**	**15.9961**	**20.6564**	**11.5943**	**1**
PSNR	23.0941	20.5736	38.1448	27.4989	32.1592	23.0941	1

**MSE**	**9102.2756**	**16253.6943**	**182.7283**	**3374.5736**	**1037.3886**	**9102.2756**	**1**
NMSE	0.0915	0.1677	0.0044	0.0341	0.0127	0.0915	1
RMS1	75.2972	103.0272	11.6934	44.2897	24.8263	75.2972	1
**PRDN1**	**28.3196**	**38.5953**	**5.4360**	**17.0167**	**10.0874**	**28.3196**	**1**
**MAX**	**664.1331**	**858.5541**	**67.4966**	**390.0220**	**195.4502**	**664.1331**	**1**
NMAX	28.4667	37.2916	3.6399	16.6038	8.8161	28.4667	1
**STDERR**	**75.3047**	**103.0375**	**11.6946**	**44.2941**	**24.8288**	**75.3047**	**1**
PAD	0.0011	0.0012	0.0002	0.0008	0.0005	0.0011	1
kappa	0.2674	0.4827	0.0102	0.0987	0.0348	0.2674	1
WWPRD	41.6217	51.2370	12.6075	29.1768	20.1287	41.6217	2
WEDD	28.5480	38.1496	3.8113	17.0078	9.2609	28.5480	2
MSEWPRD WSNA	36.6444	43.4268	13.5918	27.3532	20.1791	36.6444	2
MSEWPRD RMWSE	11.7384	15.8546	1.5654	6.9680	3.8194	11.7384	2
MSEWPRD RWSE	16.0303	21.2201	2.7006	9.8245	5.7081	16.0303	2
PE	42.2958	51.2104	14.9960	30.9513	22.5367	42.2958	2
WWPRD SWT	27.6847	37.9026	4.0311	16.3367	9.0212	27.6847	2
**WEDD SWT**	**23.3482**	**32.7345**	**2.6170**	**13.1678**	**6.7628**	**23.3482**	**2**
MSEWPRD WSNA SWT	19.4212	25.4770	4.0230	12.3197	7.5627	19.4212	2
MSEWPRD RMWSE SWT	12.0834	16.6359	1.5555	6.9919	3.7528	12.0834	2
MSEWPRD RWSE SWT	12.0834	16.6359	1.5555	6.9919	3.7528	12.0834	2
PE SWT	28.6405	38.3957	4.8176	17.5423	10.1339	28.6405	2
WDD	12.7694	15.7328	3.8303	9.1275	6.3541	12.7694	3
